# Comparing the Acceptance of Mobile Hypertension Apps for Disease Management Among Patients Versus Clinical Use Among Physicians: Cross-sectional Survey

**DOI:** 10.2196/31617

**Published:** 2022-01-06

**Authors:** Bernhard Breil, Christel Salewski, Jennifer Apolinário-Hagen

**Affiliations:** 1 Faculty of Health Care Hochschule Niederrhein, University of Applied Sciences Krefeld Germany; 2 Department of Health Psychology Faculty of Psychology University of Hagen Hagen Germany; 3 Institute of Occupational, Social and Environmental Medicine, Centre for Health and Society Faculty of Medicine Heinrich Heine University Düsseldorf Düsseldorf Germany

**Keywords:** patient acceptance of health care, mobile apps, blood pressure, mobile health, health applications, technology acceptance, patients, physicians, digital health

## Abstract

**Background:**

High blood pressure or hypertension is a vastly prevalent chronic condition among adults that can, if not appropriately treated, contribute to several life-threatening secondary diseases and events, such as stroke. In addition to first-line medication, self-management in daily life is crucial for tertiary prevention and can be supported by mobile health apps, including medication reminders. However, the prescription of medical apps is a relatively novel approach. There is limited information regarding the determinants of acceptance of such mobile health (mHealth) apps among patients as potential users and physicians as impending prescribers in direct comparison.

**Objective:**

The present study aims to investigate the determinants of the acceptance of health apps (in terms of intention to use) among patients for personal use and physicians for clinical use in German-speaking countries. Moreover, we assessed patients’ preferences regarding different delivery modes for self-care service (face-to-face services, apps, etc).

**Methods:**

Based on an extended model of the unified theory of acceptance and use of technology (UTAUT2), we performed a web-based cross-sectional survey to explore the acceptance of mHealth apps for self-management of hypertension among patients and physicians in Germany. In addition to UTAUT2 variables, we measured self-reported self-efficacy, eHealth literacy, previous experiences with health apps, perceived threat to privacy, and protection motivation as additional determinants of mHealth acceptance. Data from 163 patients and 46 physicians were analyzed using hierarchical regression and mediation analyses.

**Results:**

As expected, a significant influence of the unified theory of acceptance and use of technology (UTAUT) predictors on intentions to use hypertension apps was confirmed, especially for performance expectancy. Intention to use was moderate in patients (mean 3.5; SD 1.1; range 1-5) and physicians (mean 3.4, SD 0.9), and did not differ between both groups. Among patients, a higher degree of self-reported self-efficacy and protection motivation contributed to an increased explained variance in acceptance with *R*^2^=0.09, whereas eHealth literacy was identified as exerting a positive influence on physicians (increased *R*^2^=0.10). Furthermore, our findings indicated mediating effects of performance expectancy on the acceptance among patients but not among physicians.

**Conclusions:**

In summary, this study has identified performance expectancy as the most important determinant of the acceptance of mHealth apps for self-management of hypertension among patients and physicians. Concerning patients, we also identified mediating effects of performance expectancy on the relationships between effort expectancy and social influence and the acceptance of apps. Self-efficacy and protection motivation also contributed to an increase in the explained variance in app acceptance among patients, whereas eHealth literacy was a predictor in physicians. Our findings on additional determinants of the acceptance of health apps may help tailor educational material and self-management interventions to the needs and preferences of prospective users of hypertension apps in future research.

## Introduction

### Background

With 20 to 30 million out of approximately 82 million citizens affected in Germany alone, chronically increased blood pressure or hypertension represents a highly prevalent disease in working people, with a prevalence of 20%-25% in the age cohort of 40 to 49 years [1-3]. International studies also emphasize the role of hypertension as a leading risk factor for cardiovascular diseases as the most common cause of morbidity and mortality [4]. In addition, untreated or poorly treated chronically increased blood pressure can lead to life-threatening secondary diseases, such as heart attack or stroke. In Germany, approximately 20% of the people with high blood pressure are estimated to be unaware of their condition [5], whereas another study from the United States revealed that up to 36.2% of the concerned individuals are not aware that they suffer from hypertension [6].

Basically, hypertension results from the interaction of several factors, some of which cannot be changed, such as age or genetic disposition, whereas others can be influenced by stress, lifestyle, or health behavior [7] (eg, physical activity). Despite the availability of effective and relatively safe medication, only approximately half of the treated patients with high blood pressure are well adjusted, as indicated by epidemiological data [7]. Measuring one’s blood pressure values at home regularly is a further important prerequisite to control the disease because this promotes the patient's understanding of the disease and medication adherence [7]. Therefore, regular self-assessment or monitoring of blood pressure and a healthy lifestyle are recommended to patients [8]. All the described therapeutic approaches (self-assessment of blood pressure, taking medication regularly, and maintaining a healthy lifestyle) require a high degree of self-management by patients. Consequently, self-management represents important therapeutic potential for people suffering from hypertension [9]. However, self-management can pose high demands on patients with chronic conditions in daily life. Possible solutions include digital programs, such as disease management apps [10].

In general, mobile health (mHealth) apps are defined as digital apps on smartphones or tablets that provide health-related content and electronically record and evaluate the body data as well as behaviors of their users [11]. The features of these apps range from sending reminders via text messages to the measurement of, for example, blood pressure values via corresponding sensors. Health apps can be integrated into the everyday life of patients with hypertension and have the potential to positively influence the course of the disease in terms of improved long-term disease management, including medication reminders and monitoring [12-14]. In addition, the legal basis for integrating mHealth apps into routine care has been established in December 2019, making it possible to prescribe medical apps since October 2020, as statutory health insurance companies cover the expenses if apps are prescribed by physicians. However, despite an interest in using health apps among patients and physicians, their uptake requires considerable time to reach a population level, especially due to barriers such as the lack of knowledge about suitable options [15]. Accordingly, the acceptance of these apps, especially among patients without experience in using such apps whose functionalities vary considerably, depends highly on whether the disease-specific needs and patient preferences are met [10,12]. In addition, many studies have assessed acceptance of this technology only among patients as users or in terms of outcomes in clinical trials (eg, satisfaction), and they have not focused on early acceptance (eg, use intentions) of potential users such as patients and providers [16,17].

### Determinants of the Acceptance of Health Apps

To improve the adoption of mHealth apps among smartphone users with hypertension, it is crucial to understand the determinants of acceptance and use. An approach for predicting acceptance (ie, intention to use) and usage of innovations such as health apps is the unified theory of acceptance and use of technology (UTAUT), including its core predictors namely performance expectancy, effort expectancy, social influence, and facilitating conditions [18]. Although the UTAUT model was developed in the business context, it is being extensively used to understand acceptance of new health care technologies [19], for instance, by assessing the usage intentions of a specific health technology among patients [20,21] and physicians [17,22]. The traditional UTAUT model was extended in 2012 to include hedonistic motivation, price value, and habit, subsequently called UTAUT2 [23], and it may be especially suitable to evaluate the acceptance of apps. Given the contextual sensitivity of acceptance, several extended UTAUT models and novel assumptions on mediating effects have been proposed, such as that of effort expectancy in relation to the other 3 UTAUT determinants (eg, performance expectancy) and intention to use a technology [20]. However, the remaining challenges in applying the UTAUT model include the unclarified mediating role of performance expectancy [20] along with the scarcity of UTAUT-related studies that clearly conceptualize and investigate the individual characteristics of technology acceptance [24].

In addition to the UTAUT2 determinants, self-efficacy has also been analyzed as a factor in various studies on the acceptance of innovations in health care, including electronic patient records [25], electronic mental health interventions [16,26], and hypertension apps [21].

Patient empowerment implies that patients are being increasingly recognized as the experts of their disease. However, making informed decisions on health apps require a broad range of skills and abilities, such as eHealth literacy. In the context of health apps for self-management of chronic diseases, a positive association with acceptance appears plausible because people with higher levels of eHealth literacy are expected to be more likely to find and use effective digital support for self-management [27] and cultivate preventive health behavior [28].

In addition to the outlined UTAUT determinants, personal beliefs or concerns regarding data protection in digital apps also appear to influence the intended use of health innovations, as mentioned earlier [29,30]. In contrast to the aforementioned factors, data protection and privacy concerns represent a barrier that is not commonly included in UTAUT-based research. According to Zhang et al [20], the perceived threat to privacy negatively influences the intention to use digital apps. In Germany, Heidel and Hagist [15] also confirmed that such concerns about data privacy and security are very strong or even stronger than those in many other countries.

Besides the UTAUT, factors such as the duration and therapy of hypertension, attitudes, and evaluation of the disease also play an important role in the context of hypertension as the determinant for the use of health apps. Illness-related predictors of health app adoption can be covered by the protection motivation theory (PMT) [31]. According to the PMT, the motivation to protect arises from the assessment of a threat and possible coping strategies. Protection motivation with respect to hypertension is mainly relevant for patients because this variable reflects beliefs about one’s own health risk and not those of others. The influence of the PMT factors on the acceptance of eHealth solutions was confirmed, whereas the effect on the intention to use was found to be mediated by attitudes and moderated by age and gender [32].

However, little is known about the relative contribution of the subjective evaluation of one's disease and technology-related concerns regarding data protection aspects. Furthermore, most studies focus on 1 user group (patients or physicians), thus hampering the direct comparison of the acceptance factors between patients and health care providers (eg, prescribing medical apps).

In a prior study conducted by our work group [21], the perceived threat of the disease was identified as a significant determinant in an extended UTAUT model for hypertensive patients. However, with a total *R*^2^=0.62 for the whole model, the explained variance indicated the existence of unconsidered additional determinants. Besides the UTAUT predictors of acceptance, other variables, especially self-efficacy and perceived health threat as well as the motivation to protect oneself from this threat of the disease, may be worth investigating. Furthermore, data security concerns or perceived threats to privacy may also play a major role in the acceptance of mHealth apps [33]. In addition, our prior work did not involve the perspectives of physicians who represent the “other side” of app acceptance, namely the perspective of potential prescribers of medical apps.

Patients and physicians differ in several aspects regarding their acceptance of hypertension apps, especially based on their motivations or reasons to use these apps. For patients, the focus is on managing their own disease to avoid health deterioration [12,34]. Thus, acceptance of health apps crucially depends on whether disease-specific needs are met [12,15]. Telemedicine is the main category for any eHealth solution that is used by physicians in health care.

To date, the adoption (or acceptance) of telehealth, including mHealth apps, by physicians has been studied primarily in terms of its benefits in supporting their work, such as reduced time and effort [35], rather than the potential benefits and risks to their patients with chronic conditions. Regarding specific differences, physicians have been found to report more open attitudes with less fear of risk compared to patients in Germany [36].

Hence, the present study investigates the acceptance of health apps for managing hypertension among patients and physicians. Thus, it addresses an area of research that has not yet been exhaustively investigated across different contexts, beyond the clinical testing of hypertension apps [16]. To improve the understanding of the efficient adoption of medical apps, the perspectives of patients and physicians or providers are important. Therefore, the present study examines similarities and differences between the 2 groups. In particular, it examines beliefs and expectations regarding the use of mHealth apps. To our knowledge, this is the first study that analyzes determinants for acceptance by both user groups in Germany.

### Objectives

This study aims to complement existing research on mHealth acceptance by applying the extended UTAUT model and specifically focusing on other individual predictors related to hypertension and global user-related characteristics (eg, self-efficacy, eHealth literacy) [37], differentiated by personal use (patients) and clinical use (physicians). This is one of the few studies that investigates a possible disease-specific influence, examining patient and physician acceptance simultaneously to determine similarities and differences between these complementary user groups of mHealth apps [38]. Another goal is to explore the assumed underlying mechanisms (ie, mediator effects) in the relationships of the proposed extended UTAUT2 model [23], particularly regarding the role of performance expectancy. Although performance expectancy was only investigated as a predictor with a direct influence on the intention to use in the original UTAUT model, the extended UTAUT model for health care developed by Zhang et al [20] also found that performance expectancy plays a mediating role. Therefore, we explored the potential mediating effects of this construct because they are not yet fully understood.

Based on prior research and theoretical considerations, we propose the following research questions to analyze the predictors of acceptance.

1. Which factors determine the acceptance (intention to use) of medical apps for self-management of hypertension among patients (personal use) and physicians (clinical use)?
2. Does the acceptance (intention to use) of hypertension apps differ between patients (personal use) and physicians (clinical use) based on varying cognitive attitudes, beliefs, and expectations (eg, UTAUT determinants like performance expectancy), and affective attitudes or beliefs (eg, concerns, perceived privacy threat, hedonic motivation)?
3. Does performance expectancy mediate the relationship between the other UTAUT determinants and acceptance among patients?

## Methods

### Study Design and Participant Recruitment

This study was designed as a cross-sectional web-based questionnaire study using the Unipark software (Questback Enterprise Feedback Suite Questionnaire, version 2019). Inclusion criteria were a minimum age of 18 years, fluency in written German, and being either a patient with self-reported hypertension (survey version 1, personal use) or a practicing physician regardless of specialty (survey version 2, clinical use). As some of the predictors were operationalized differently or not applicable to the 2 target groups (eg, duration of disease), 2 versions of the survey with different item sets were created and displayed after using an initial filter question (see Multimedia Appendix 1 for the version concerning the user group). Prior to data collection, a pretest with 7 people (4 patients and 3 physicians) was conducted. After this pretest, only semantic adjustments were made in the instructional texts, as some terms were not comprehensible to laypersons (eg, hypertension). An a priori power analysis was conducted to calculate the required sample size for multiple linear regression analyses with a maximum of 14 predictors and an expected moderate effect of f^2^=0.15, resulting in an estimated sample size of 151 persons (with an α error probability of .05 and a power of 0.9) The data were collected anonymously between September 14, 2019, and October 31, 2019. Participation was voluntary. The overall completion time was 10 to 15 minutes on average. The 2 target groups (adult patients at least 18 years old with hypertension and physicians) were recruited primarily via social networks (eg, XING, Facebook, and Twitter), personal invitations in private and work environments (including medical conferences), emails, contributions in self-help forums and interest groups, and a university website. There was no monetary compensation. Undergraduate psychology students enrolled at the University of Hagen, a distance-learning university (patients or physicians in a second degree program), could be compensated with study credits via a virtual lab. As an incentive to participate in the study, a summary of the aggregated study results upon completion of the whole study was offered. The study was approved by the ethics committee of the University of Hagen prior to data collection (NR. EA_140_2019).

### Measures

#### Acceptance

The dependent variable for patients and physicians is the acceptance of health apps, namely the intention to use health apps for managing chronic diseases (ie, hypertension) either as patients or physicians. This outcome was operationalized differently for both user groups. This study addressed the acceptance of hypertension apps in general, mainly in terms of the intention to use them (ie, not specific existing apps). For patients, the intention to use apps was assessed in line with a study by Breil et al [21] with 3 variables on a 5-level Likert scale from “do not agree at all” to “fully agree.” Among physicians, acceptance was operationalized as their intention to use health apps in their clinical practice and not manage their health in contrast to patients (ie, “I could imagine incorporating health apps into my work”) and whether they would recommend such health apps in general to their patients (“I would recommend patients use health apps”). For physicians, 4 items in a 5-point Likert scale were used to determine their intention to use health apps in their own work. [17].

**Figure 1:**
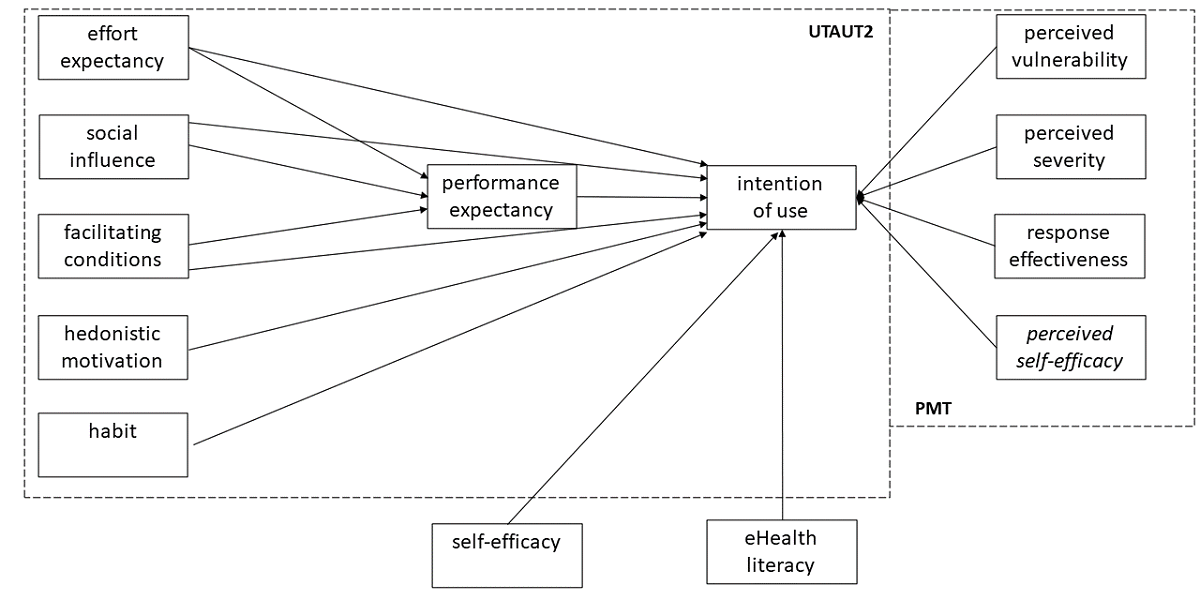
Research model depicting the acceptance of hypertension apps by patients. This study analyzes the influence of the determinants in the adapted UTAUT2 model and the protection motivation theory on the intention of using hypertension apps in addition to self-efficacy and eHealth literacy. eHealth: electronic health; PMT: protection motivation theory; UTAUT2: unified theory of acceptance and use of technology.

As illustrated in Figure 1, we have adapted and extended the UTAUT2 research model. Only the price value in the UTAUT2 model was not considered due to the specific context (statutory health system in Germany) and the low familiarity with digital health in Germany [39]. The research model for physicians omits the PMT factors because this theory is only applicable to patients and not to physicians in their role as health care providers.

#### UTAUT2 Determinants of Acceptance

The operationalization of the UTAUT and UTAUT2 variables was based on the work of Zhang et al [20] with translations according to Hennemann et al [16], Breil et al [21], and Harboth and Pape [40]. The constructs hedonistic motivation and habit (UTAUT2) were additionally included in this study [23]. All UTAUT items were assessed on a 5-point Likert scale from “do not agree at all” to “fully agree.” The Cronbach α was in the acceptable to good range for all scales between .74 and .91, except for facilitating conditions for physicians (Cronbach α=.61). The complete questionnaire can be found in Multimedia Appendix 1.

#### Further Determinants of Acceptance

Self-efficacy was assessed using the General Self-Efficacy Short Scale with 3 items on a 5-point Likert scale ranging from “fully disagree” to “fully agree” [41]. The internal consistency of the scale was good (Cronbach α=.83).

#### Technology-Related Determinants

Norman et al [42] define eHealth literacy as the ability to search, find, understand, and evaluate health information from electronic sources and to use the knowledge thus gained to address or solve a health problem. eHealth literacy was operationalized with 8 items on a 5-point Likert scale ranging from “fully disagree” to “fully agree” using the electronic health literacy scale (eHEALS) [42] in German according of Soellner et al [43] (eg, “I know how to use the health information I find on the internet to help me“). Internal consistency of the scale was good with a Cronbach α=.89.

Perceived threat to privacy was operationalized according to Zhang et al [20]. Instead of the 7-point Likert scale, a 5-level Likert scale ranging from “strongly disagree” to “strongly agree” was used [20].

### Experience With eHealth

Some studies have shown a significant positive influence of prior experience with web-based services on the acceptance of eHealth [44,45]. Even though most of the apps in the cited prior work were related to mental health services, a positive effect was also expected for hypertension apps in the present study. According to Venkatesh et al [23], experience is also a relevant moderating factor in other contexts; therefore, experience with health apps was also investigated in this study.

Contrary to the eHealth experience data collected for both user groups with the same items, the items for physicians were specifically adapted to the context of smartphone usage in a professional context with respect to clinical practice. The items were based on Albrecht et al [35] and included the use of apps in general (dummy coded, with 1=yes and 0=no), type of usage (professional, private), and types of the activities and concerns that prevent them from using smartphone apps.

In addition to the aforementioned constructs, the participants' age, gender, highest educational attainment, as well as the current country of residence and region (urban vs rural) were included as the control variables.

### Health-Related Determinants

Information on the patients' own high blood pressure was obtained with 3 items. The durations of the disease and medication intake were recorded metrically in years. Comorbid diseases were chosen based on the list of chronic diseases provided by the Robert Koch Institute (ie, the German Higher Federal Authority for Infectious Diseases) [46] (multiple answers were possible).

### Protection Motivation

Protection motivation was measured using the PMT questionnaire that is based on the following components [32]: Perceived vulnerability describes the probability of the occurrence of an illness-related event. Especially in the context of hypertension, several risk factors can be identified such as higher age as well as modifiable lifestyle factors, such as malnutrition and less physical activity. Perceived severity describes the extent to which a depicted event is perceived as harmful. Response effectiveness refers to specific protective behavior, such as the use of health apps and assessment of their effectiveness. As a fourth component*,* self-efficacy is investigated in terms of the skills needed to perform the protective behavior.

The PMT variables were operationalized according to Guo et al [32] with the 4 components, namely perceived vulnerability, perceived severity, response efficacy, and perceived self-efficacy. According to the PMT, protection motivation results from the subjective assessment of a threat and possible coping strategies. To ensure a concrete reference to high blood pressure, the sentence “Possible consequences of high blood pressure are various cardiovascular diseases (including heart attack, stroke), retinal damage, kidney damage, etc” was placed at the beginning of the questions.

The influence of the PMT variables was considered relevant only for the patients participating in this study and was thus not investigated among physicians.

### Statistical Analysis

Only completed surveys were extracted from Unipark and analyzed (due to option of consent withdrawal by dropping out). The influence of the different UTAUT and PMT predictors on the intention to use self-management apps for hypertension (ie, acceptance) was computed using simple linear and multiple hierarchical regression analyses separately for the 2 user groups (ie, patients and physicians). The prerequisites for the parametric tests were examined and found to be sufficiently applicable.

To investigate the relative influence of variables, the significant determinants in the simple regression analyses were transferred to an overall model in 4 blocks, as shown in Table 3. This was done separately for both user groups due to the different determinants. First, the patients were considered. In multiple hierarchical regression, all significant single predictors were included in blocks. As the UTAUT determinants have already explained up to 70% of the intention to use in prior research [23], the UTAUT factors in this study were included as the first block or step of the hierarchical linear regression model followed by self-efficacy in the second block. In the third block, eHealth literacy was included, and the fourth block contained the items from the perceived threat to privacy. The fifth and last block comprised the 4 factors from the PMT. For each block, the increase in the coefficient of determination (∆*R*^2^) was determined. In line with Zhang et al [20], the effect of the other factors such as self-efficacy, privacy concerns, and factors from the PMT were analyzed in the subsequent blocks [20]. Differences in the acceptance scores between the 2 user groups were calculated through *t* tests for independent samples.

In addition to analyzing the intention to use health apps, we assessed preferences in terms of the willingness of patients to use health apps compared to face-to-face consultations with physicians, self-help groups, or internet-based information for managing high blood pressure.

To test the assumed mediation effects, 3 regression analyses were conducted for each of these assumptions. The first step of the regression model tested whether the predictor variables influenced the mediator variable performance expectancy. In the second step of the regression model, the direct effect of predictor variables on the dependent variable was determined, as already confirmed for all the 3 variables in the prior step. In the third step, the indirect effect was determined.

The analyses were performed using SPSS Statistics (version 25, IBM Corp). Conditional effects, especially related to the moderation hypotheses, as well as the indirect effects and the associated mediation hypotheses were calculated with PROCESS (version 3.4), a macro in SPSS [47]. Bootstrapping analysis was performed with 5000 bias-corrected samples to calculate the total direct and indirect effects of the variables. Hypotheses were tested twofold at α<.05.

## Results

### Sample Characteristics

In the period mentioned earlier, 337 people accessed the internet-based survey, with 212 people giving their consent and completing the survey. This corresponds to a completion rate of 62.9%. However, 13 respondents did not start the survey at all, and 112 people did not finish it. Participants dropped out mainly because of not providing informed consent (26/337, 7.7%), not stating to which of the 2 user groups they belonged (42/337, 12.5%), and not providing demographic information (19/337, 5.6%). Moreover, 3 respondents were excluded after reviewing the raw data, as they had not answered the initial question regarding the user group (patient or physician) and thus had not answered the user group–specific questions (eg, on PMT variables).

The sample for the data analysis consisted of 209 participants including 163 patients and 46 physicians. The mean age was 35 years (mean 35.3 [SD 13.8] years), with slight differences between the user groups, as shown in Table 1. More women (126/209, 60.3%) participated in the survey than men. Differences between the user groups were apparent in terms of educational attainment, as shown in Table 1.

**Table 1:** Demographic characteristics.

Characteristics		Total sample (N=209)	Patients (n=163)	Physicians (n=46)
**Age (years)**				
	Mean (SD)	35.26 (13.8)	35.53 (14.9)	34.28 (8.6)
	Range (median)	18-79 (33)	18-76 (32)	18-53 (34)
**Sex, n (%)**				
	Female	126 (60.3)	98 (60.1)	28 (60.9)
	Male	82 (39.2)	64 (39.3)	18 (39.1)
	Not mentioned	1 (0.5)	1 (0.6)	0 (0)
**Education, n (%)**				
	High school graduation	114 (54.5)	104 (63.8)	10 (21.7)
	University degree	95 (45.5)	59 (36.2)	36 (78.3)

As shown in Table 2, 129 of the 209 participants (61.7%) stated that they already had experience using mobile health apps. Here, patients (103/163, 63.2%) differed only slightly from physicians (26/46, 56.5%) in terms of experience. Both groups had been using health apps for approximately 2.5 years on average (SD 2.9 and 3.1, respectively). There were clear differences in the way they used the apps. “Vital value measurements” (51/163, 31% vs 7/46, 15.2%) and “memories” (46/163, 28.2% vs 8/46, 17.4%) were used more frequently by patients, whereas every physician cited “Search for information” as the reason for use. Table 2 shows previous experience with health apps differentiated by user group. Under “Other use,” patients indicated that they also used “apps for measuring movement or physical activity (steps),” “menstruation cycle apps,” and “pregnancy apps.”

**Table 2:** Experience using electronic health apps.

			Total sample (N=209)	Patients (n=163)	Physicians (n=46)
**Experience with health apps, n (%)**					
	Yes		129 (61.7)	103 (63.2)	26 (56.5)
	No		74 (35.4)	55 (33.7)	19 (41.3)
	Not specified		6 (2.9)	5 (3.1)	1 (2.2)
**Purpose of using apps, n (%)**					
	Vital signs measurement		58 (27.8)	51 (31.3)	7 (15.2)
	Reminder		54 (25.8)	46 (28.2)	8 (17.4)
	Documentation		47 (22.5)	37 (22.7)	10 (21.7)
	Electronic communication		50 (23.9)	35 (21.5)	15 (32.6)
	Search for information		68 (32.5)	45 (27.6)	23 (50)
	Relaxation		53 (25.4)	40 (24.5)	13 (28.3)
	Other		24 (11.5)	17 (10.4)	7 (15.2)
**App selection basis, n (%)**					
	Searched or found by self		126 (60.3)	103 (63.2)	23 (50)
	Recommendation from friends		40 (19.1)	34 (20.9)	6 (13)
		Recommendation from physicians	33 (15.8)	15 (9.2)	18 (39.1)
		Advertising	16 (7.7)	15 (9.2)	1 (2.2)
		Other	10 (4.8)	N/A^a^	N/A

^a^N/A: not applicable.

There were differences not only in the purpose, way, and frequency in which apps were used but also in the search and selection of apps. In both groups, apps were mainly searched for or found by the users themselves (98/163 patients and 28/46 physicians, 60.3%). Recommendations from friends were relevant for 20.9% (34/163) of patients and 13% (6/46) of physicians. Advertising was a reason for the selection of health apps for 9.9% (16/163) patients but only for 2.2% (1/46) of the physicians.

#### Patients’ Preferences

Mobile health apps were stated as the second most preferred option to support hypertension management by 30.7% (50/163) of the patients. Only direct-contact physician care was regarded as more preferable for 36.8% (60) of the patients. In contrast, medical care via the internet 9.2% (15, 9.2%) and local face-to-face groups such as support groups (10, 6.1%) were the options mentioned much less frequently.

#### Previous Use of Smartphones by Physicians

Most physicians (35/46, 76.1%) reported using their smartphone for job-related electronic communication with patients or other professionals through email, chat, or messenger functions. The internet was also frequently used for searching literature in journals or databases by 60.9% (28) of the physicians. Other frequent responses included information on medication and treatment options given by 43.5% (20) and access to training content by 37% (17) of the physicians. Requests for laboratory tests (4, 8.7%) and access to patient records (5, 10.9%) were less frequently reported.

#### Physicians’ Concerns When Using Health Apps

Physicians were asked about their concerns when using health apps. The main concerns were about the security of patient data (37/46, 80.4%), followed by the trustworthiness of content (23, 50%) and technical reliability of software (20, 43.5%). Concerns about hygiene were mentioned by only 23.9% (11) of the physicians. Concerns about reimbursement by German statutory health insurance companies (3, 6.5%), lack of or limited options for access by patients (4, 8.7%), and poor acceptance by patients (8, 17.4%) were relatively low.

### Preliminary Analyses

Preliminary analyses were conducted to select significant determinants for the hierarchical regression model for patients. In simple linear regression, performance expectancy had a positive effect on the intention to use hypertension apps, with the highest explained variance of UTAUT determinants (*R*^2^=0.44; β=.66; *P*<.001). There were also significant positive influences of effort expectancy (*R*^2^=0.25;β=.50; *P*<.001), social influence (*R*^2^=0.13; β=.36; *P*<.001), facilitating conditions (*R*^2^=0.23; β=.48; *P*<.001), hedonistic motivation (*R*^2^=0.15; β=.39; *P*<.001), and habit (*R*^2^=0.13; β=.36; *P*<.001) on the intention to use. Subsequently, all significant UTAUT predictors were included in a multiple hierarchical model (Table 3). Of the 6 UTAUT factors, only performance expectancy had a statistically significant influence on patients (t_156_=6.27, *P*<.001). Except for performance expectancy, the other predictors did not contribute significantly to the overall model of acceptance. Multimedia Appendix 2 presents the entire linear model.

Single regressions were conducted for analyzing the influence of the PMT variables. Perceived vulnerability (*R*^2^=0.11; β=.33; *P*<.001), perceived severity (*R*^2^=0.07; β=.27; *P*<.001), response efficacy (*R*^2^=0.29; β=.54; *P*<.001), and perceived self-efficacy (*R*^2^ =0.31; β=.56; *P*<.001) positively influenced the intention to use hypertension apps. In the multiple regression model, all factors except for perceived severity made a significant contribution (*R*^2^=0.42). Multimedia Appendix 3 shows the multiple linear regression model of the PMT with all 4 factors and confidence intervals for β.

Next, simple regression analyses were also conducted for the physician user group. Performance expectancy had a positive effect on the intention to use (*R*^2^=0.21; β=.46; *P*<.01). In contrast, effort expectancy, social influence, facilitating conditions (UTAUT1), hedonistic motivation, and habit (UTAUT2) did not prove significant in the simple regression model (*P*>.05).

### Main Results

#### Research Question 1: Acceptance Determinants for Patients and Physicians

For the overall model regarding patients, the explained variance was *R*^2^=0.56 (*F*_15_= 12.53, *P*<.01). Of the 15 predictors in the 5 blocks, only 3 predictors were significant in the hierarchical regression (last step). Performance expectancy significantly contributed to the prediction of the intention to use hypertension apps (*R*^2^=0.47). Among the remaining variables, only self-efficacy (∆*R*^2^=0.02) and protection motivation in terms of the PMT variables (∆*R*^2^=0.07) made significant contributions to the explained variance of the overall model for the patient group, as shown in Table 3.

Simple regression analyses showed that previous experience with health apps contributed to the acceptance of health apps for managing hypertension among patients (*R*^2^=0.20, *P*<.01).

Determinants of hypertension app acceptance among physicians were then examined, as demonstrated in Table 4. The UTAUT variables form the first block. Among physicians, previous experience with health apps was not significant.

**Table 3:** Overall model of the determinants for the intention to use hypertension apps in patients (n=162).

Predictor		B^a^	SE	95% CI	β	*P* value	Δ*R*^2^	
Constant		–1.85	1.58	–4.98 to 1.28		.25		
**UTAUT^b^ determinants**							0.47	
	Performance expectancy	0.48	0.11	0.26 to 0.70	.42	<.001		
	Effort expectancy	–0.03	0.10	–0.23 to 0.16	–.04	.73		
	Social influence	–0.02	0.09	–0.19 to 0.15	–.02	.82		
	Facilitating conditions	0.07	0.09	–0.11 to 0.24	.06	.46		
	Hedonistic motivation	0.13	0.08	–0.03 to 0.28	.11	.10		
	Habit	–0.10	0.08	–0.27 to 0.07	–.09	.24		
**Self-efficacy**								0.02
	Self-efficacy expectation	0.19	0.10	0.00 to 0.38	.13	.05		
eHealth^c^ literacy		–0.01	0.04	–0.10 to 0.07	.06	.73	<0.01	
**Threat of privacy**								<0.01
	Usage for other purpose	0.19	0.23	–0.26 to 0.65	.06	.40		
	Loss/leakage of personal data	–0.10	0.25	–0.59 to 0.40	–.03	.70		
	Misuse of personal data by criminals	–0.30	0.23	–0.75 to 0.16	–.10	.20		
**Protection motivation**								0.07
	Perceived vulnerability	0.24	0.08	0.09-0.39	.20	<.001		
	Perceived severity	0.03	0.08	-0.12-0.18	.03	.67		
	Response efficacy	0.09	0.08	-0.08-0.18	.09	.29		
	Perceived self-efficacy	0.19	0.11	-0.03-0.40	.17	.09		

^a^B: unstandardized β.

^b^UTAUT: unified theory of acceptance and use of technology.

^c^eHealth: electronic health.

**Table 4:** Hierarchical regression model of the determinants for the intention to use in physicians (n=46)^a^.

Predictor		B^b^	SE	95% CI	β	*P* value	Δ*R*^2^	
Constant		4.31	4.30	–4.41 to 13.03	0	.32		
**UTAUT^c^ determinants**								0.27
	Performance expectancy	0.76	0.28	0.20 to 1.32	.54	.01		
	Effort expectancy	–0.33	0.20	–0.74 to 0.07	–.29	.11		
	Social influence	–0.29	0.28	–0.86 to 0.28	–.20	.30		
	Facilitating conditions	–0.04	0.19	–0.43 to 0.34	–.04	.82		
	Hedonistic motivation	0.05	0.22	–0.40 to 0.51	.04	.82		
	Habit	0.06	0.22	–0.39 to 0.51	.06	.78		
eHealth literacy		0.30	0.11	0.07 to 0.53	.41	.01	0.10	
**Threat of privacy**								0.05
	Usage for other purposes	0.45	0.57	–0.71 to 1.62	.12	.43		
	Loss or leakage of personal data	–1.03	0.60	–2.25 to 0.19	–.29	.09		
	Misuse of personal data by criminals	0.38	0.50	–0.64 to 1.39	.11	.45		

^a^Data concerning self-efficacy and PMT were collected only for patients.

^b^B: unstandardized β.

^c^UTAUT: unified theory of acceptance and use of technology.

#### Research Question 2: Differences Between Patients’ and Physicians’ Acceptance of Hypertension Apps

To test for differences between physicians and patients, all significant influences were transferred to a multiple regression model for each group and included in blocks. Self-efficacy had a significant influence only in the patient group and was therefore omitted in the multiple regression model. In the second block, eHealth literacy was included. The third and last block involved the perceived threat to privacy. For each block, the increase in the coefficient of determination (∆*R*^2^) was identified.

In the case of the patients, only 3 out of the 15 predictors in the 5 blocks were significant in the hierarchical regression. Performance expectancy contributed significantly to the prediction with *R*^2^=0.47. Beyond that, only self-efficacy (∆*R*^2^=0.02) and PMT (∆*R*^2^=0.07) made significant contributions.

For physicians, all significant individual predictors were also included block wise in the multiple hierarchical regression. For the overall model, the coefficient of determination was *R*^2^=0.42. The UTAUT2 factors explained under one-third of the variance explained by the overall model, with *R*^2^=0.27. A further 10% increment in *R*^2^ resulted from the addition of eHealth literacy and another 5% by accounting for privacy threat.

Direct comparison shows that the *R*^2^ for patients is slightly higher and is largely determined by UTAUT2; therefore, the addition of other determinants resulted in a comparatively small increase in *R*^2^. For physicians, the influence of factors outside of the UTAUT factors (eHEALS and privacy) is stronger.

#### Research Question 3: Mediation Effects in the Extended UTAUT Model for Patients

**Figure 2:**
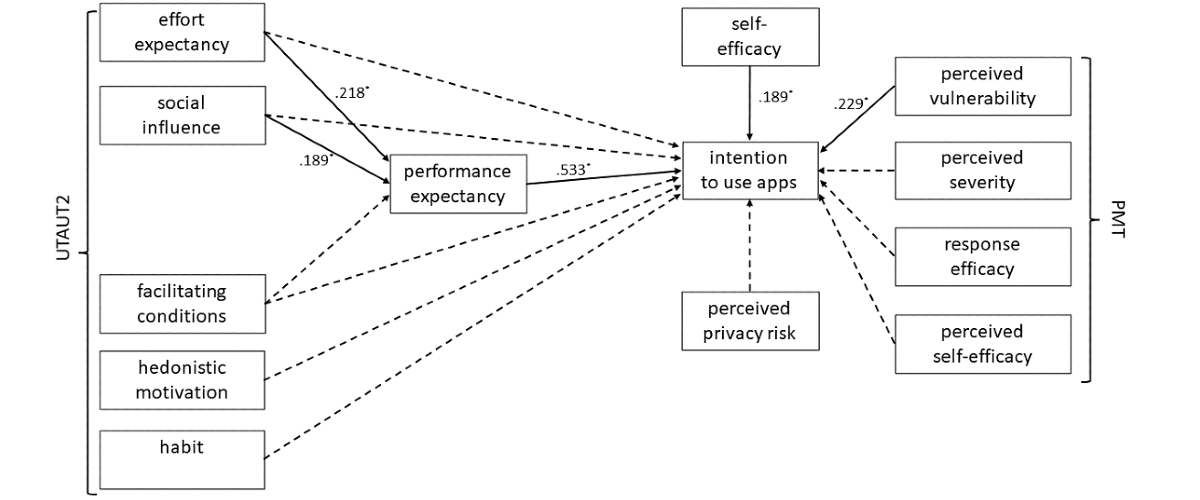
Overall model showing the determinants of the intention to use hypertension apps in patients. Significant influence is shown with solid lines and corresponding beta values; influences that were investigated but not significant are shown with dashed lines. PMT: protection motivation theory; UTAUT2: unified theory of acceptance and use of technology.

In line with the UTAUT assumptions, the UTAUT predictors effort expectancy, social influence, and facilitating conditions exerted a significant direct influence on performance expectancy*,* as illustrated in Figure 2. Performance expectancy had a significant direct effect on the intention to use hypertension apps and mediated the relationship between effort expectancy and intention to use (95% CI 0.04-0.23) as well as the relationship between social influence and intention to use (95% CI 0.04- 0.19)*.*

The other 3 factors having a significant influence on the intention to use hypertension apps in the simple regression analyses (preliminary analyses) were used as covariates in this model. Table 5 presents the direct effects on the mediator performance expectancy as well as the direct and indirect effects on the intention to use hypertension apps.

**Table 5:** Overall model of determinants of intention to use among patients.

Predictor		Performance expectancy		Intention to use		
		β	*P* value	β	*P* value	95% CI
**Effort expectancy**						
	Direct effect	.22	.004	–.02	.82	–0.21 to 0.17
	Indirect effect	N/A^a^	N/A	.12		0.04 to 0.23
	Total effect	.22	.004	.09	.36	–0.11 to 0.30
**Social influence**						
	Direct effect	.19	.004	.00	.98	–0.17 to 0.17
	Indirect effect	N/A	N/A	.10		0.04 to 0.19
	Total effect	.19	.004	.10	.27	–0.08 to 0.27
**Facilitating conditions**						
	Direct effect	.01	.93	.08	.38	–0.10 to 0.25
	Indirect effect	N/A	N/A	.00		–0.10 to 0.08
	Total effect	.01	.93	.08	.40	–0.11 to 0.27

^a^N/A: not applicable.

## Discussion

The aim of this study was to determine the subjective factors that influence the acceptance of hypertension apps among patients and physicians in Germany. In addition to the UTAUT determinants that have already been investigated in health care research, protection motivation, threat to privacy, and self-efficacy expectations were also considered as further influencing factors on the intention to use hypertension apps.

### Principal Findings and Comparison With Prior Work

As expected, a significant influence of performance expectancy on the acceptance of hypertension apps was found among patients and physicians. Among patients, self-efficacy and protection motivation, including perceived threat, further contributed to an increase in the explained variance of the extended UTAUT2 model.

The differences between the 2 user groups indicated that several factors had a statistically significant influence only in patients, such as self-efficacy. In the physician group, only performance expectancy proved significant. In addition to the UTAUT factors, a significant influence of eHealth literacy was identified only in physicians. Potentially, physicians had a more differentiated understanding of the meaning of eHealth literacy and were thus more critical in assessing their own skills than patients [48].

Another goal of this study was to gain insights into the role of performance expectancy as a mediating variable. Although numerous studies on the UTAUT model [49] have confirmed a direct influence of performance expectancy, they have not clarified whether this variable also mediates the influence of beliefs on the intention to use mHealth apps. Hence, a methodologically added value to the UTAUT2 model in this study can be observed in the demonstrated mediator role of performance expectancy, as shown earlier by Zhang et al [20]. Specifically, our study demonstrated the mediating effects of performance expectancy in the relationship between effort expectancy as well as social influence and the intention to use hypertension apps in patients. We also identified a direct effect of perceived vulnerability. Thus, the strong influence of performance expectancy and its mediating role may explain why the other UTAUT factors had no statistically relevant influence in the hierarchical linear model.

In contrast, the expected moderating effects of previous experience with health apps could not be identified in the group of patients, which should be interpreted considering the web-based survey and self-selection bias.

Contrary to our assumptions and previous research indicating data security concerns as a major barrier to using health apps [50], perceived threat to privacy had no significant influence on the acceptance of hypertension apps in our study. Potentially, the sample was already aware of certified disease management apps approved by statutory insurance companies and other trusted sources in Germany.

The explained variance of the UTAUT determinants that we applied in the extended UTAUT2 model in this study was *R*^2^=0.47. In comparison, for all determinants, the explained variance regarding app acceptance by patients was only slightly higher with *R*^2^=0.56 (ie, all 5 blocks in the regression model). This finding corresponds, for instance, to a study by Dou et al [51], which obtained an *R*^2^ of 0.412 based on various determinants regarding the intention to use apps for self-management of chronic diseases.

More specifically, the predictive value in terms of the explained variance of the proposed determinants of acceptance is comparable to our previous study that served as a basis for this survey [21], which is interesting because of the integration of more illness-related variables in this study. In our related study [21], performance expectancy and effort expectancy proved to be significant predictors, explaining approximately 50% of the variance in the acceptance scores among patients. For direct comparison, the Illness Perception Questionnaire [52] that we used only in our previous study may have been a better choice for capturing the disease-specific acceptance of apps, as its contribution to the total variance was higher compared to the PMT factors. Instead, vulnerability was the only significant variable of the PMT block in the multiple regression model used in this study. Remarkably, threat to privacy, which had not yet been surveyed in our preliminary study [21], was thought to be another significant determinant in this study. However, we could not confirm this assumption, which may have been due to the rather young adults constituting our patient and physician samples.

Given the unexplained variance, patients’ perspectives, especially regarding unmet needs and preferences, could be further explored using mixed methods and qualitative research methods. Accordingly, a qualitative study by Morrissey et al [53] also highlighted concerns regarding the risks of health apps used to improve medication adherence and the need for promote eHealth literacy among hypertensive patients. Regarding the real-world assessment of apps, a mixed methods study by Allessa et al [34] showed that apps for self-management of hypertension can be functional and acceptable to users, but they can also be considerably improved through training [34], which corresponds to UTAUT determinants like performance and effort expectancy as well as facilitating conditions.

Interestingly, one-third of the patients in our study stated that they preferred using health apps over physician contact and face-to-face self-help groups to manage hypertension. This finding indicates that for a relevant proportion of the patients, self-management via health apps can be the first choice, which can be seen as a starting point for the implementation and additional provision of medical apps. Nonetheless, in line with prior research [54,55], most patients in our study preferred personal contact with physicians over digital self-help using hypertension apps. Hence, further research is needed to determine how to increase the adoption of mobile solutions in conjunction with traditional face-to-face health care services (eg, blended or stepped care approaches). Regular blood pressure measurement supported by apps may help bridge the gap between the medical and lay perspectives of optimal and personalized hypertension treatments in practice and promote more effective disease management in the long run [56].

Overall, this corresponds to a study by Edwards et al [57] documenting considerable interest in using telemedicine services like apps among patients with chronic diseases, regardless of their health status, access difficulties, as well as age and many other sociodemographic factors.

### Limitations

The present study is subject to several limitations. First, when considering the demographic distribution of the sample, various limitations apply that may help explain some of the consistent findings. For instance, the mean age of the physicians was considerably less at 34 years (mean 34.3 [SD 8.6] years, median 34 years) compared to all the physicians in Germany. According to the Federal Statistical Office, the average age of the physicians was 48 years in 2017 [58]. The respondents were thus considerably younger and therefore not representative of physicians in Germany. The patients in our sample were also relatively young, with the average age being 36 years (mean 35.5 [SD 14.9] years, median 32 years), and may therefore not necessarily reflect the views of most patients with hypertension, especially in terms of the acceptance and use of disease management apps. Further limitations concern the rather high educational level of the patients, with one-third holding an academic degree. Nonetheless, this group may represent a subgroup of patients that have been recently diagnosed and may thus be easily reached for prevention and health promotion initiatives.

Second, although the total sample size of 209 individuals was sufficiently powered for the conducted hierarchical regression analyses, this only applies to the analyses that concern the entire sample. Although the group of patients was sufficiently large with 163 participants, the group of physicians was relatively small (46 physicians), thus making it more difficult to identify effects. Despite including fewer determinants for the regression model measuring the acceptance of apps among physicians compared to the patient group, it would have been necessary to have a considerably higher number of physicians as participants. However, physicians are usually difficult to recruit via social media. In addition, the recruitment period of 1 month was considerably short. Therefore, the small sample size of the physician group is a major limitation.

### Implications

Implications derived from this study, based on several significant and insignificant findings, especially concern the further extension and adaptation of the UTAUT2 model in the context of chronic diseases. Holden and Karsh [59] note that it is important to continuously adapt the acceptance models for the use of telemedicine, including health apps to mirror ongoing technological advances. Thus, future research may also consider further barriers to using hypertension apps. According to Schreiweis et al [60], potential barriers and drivers for eHealth applications can be divided into individual, organizational, and technical factors. In the current study, organizational and environmental [33] policy aspects were not investigated. Instead, we focused on individual acceptance-related factors such as eHealth literacy and beliefs such as performance expectancy as well as motivational factors (eg, hedonic motivation, protection motivation). Other potentially relevant aspects such as training of physicians or information on the availability of eHealth services [51] could also be considered in upcoming studies with a broader scope on the validation of an extended UTAUT2 model for assessing the acceptance of disease management apps among patients and physicians [48].

Another variable to consider in the investigation of hypertension apps may be resistance to change among patients [51]. Given the high prevalence of hypertension, it should be considered that there is a long-term demand for treatment support and at least a relevant proportion of patients indicates a preference for health apps [61]. However, other studies with hypertensive patients indicated ambivalent views on self-management apps [53]. Patient preferences for hypertension apps may also vary depending on the different features of such apps. For instance, some studies found reminders and personalization to be important features of hypertension apps [62]. In future studies, a differentiated assessment of app features, including trade-offs between preferred features, should be considered.

Given the forecast of the German National Association of Statutory Health Insurance, physicians state that the demand for medical care will increase by 2% by 2030, whereas the supply of medical care, especially in rural areas, will continue to decline [63]; health apps could be a solution accepted by relevant target groups, as this study has indicated. Nevertheless, knowledge on the most important determinants of mHealth acceptance is required to tailor information as well as interventions to the needs and preferences of future users. In this context, it is important to note that our study was conducted shortly before the global outbreak of the COVID-19 pandemic and the introduction of the directory for digital health applications in Germany (German name: Digitale Gesundheitsanwendung [DiGA]). DiGA are defined as low-risk medical products based on digital technologies that are intended, for example, to detect or alleviate illnesses or to support diagnosis using apps or browser-based applications. The DiGA directory [64] lists all the DiGA that have successfully undergone the assessment procedure that is regulated by the Federal Institute for Drugs and Medical Devices (German name: Bundesinstitut für Arzneimittel und Medizinprodukte). Interestingly, there is no app at present (as of October 2021) for hypertension management in the recently introduced DiGA directory among the 24 listed medical apps, which may change soon. In future, the provision of certified hypertension apps may change the views and uptake of such apps in health care.

In addition, it may be important for future research to consider the connection of remote and personal treatment assistance in the management of hypertension in terms of blended or hybrid treatments [11]. Transparent quality criteria represent another key strategy for the adoption of health apps. However, the quality of commercially or publicly available apps for hypertension management has been classified as overall poor [55]. Associations between the relevant features and outcomes of hypertension apps also remain inconclusive [65]. Therefore, implementation strategies and advances in (digital) health policy, such as the DiGA registry in Germany, are important steps to increase the dissemination of quality-approved medical apps for chronic diseases. With the ongoing diffusion of medical apps into routine care, research on the acceptance and use of these apps is required on a longitudinal basis.

### Conclusions

In summary, this study identified several relevant determinants of the acceptance of hypertension apps among patients and physicians. The ongoing implementation of health apps into routine care and the COVID-19 pandemic emphasize the importance of acceptance-related research on disease management apps. One possible strategy is the targeted collection and prioritization of patient requirements [66]. Another strategy may be to increase the awareness of quality-approved self-management apps for hypertension through targeted information campaigns and training of physicians, such as general practitioners, which can be grounded on acceptance-based surveys like the present study.

Thus, the main contribution of this study lies in the identification of additional disease-related, context-sensitive determinants of the intention to use hypertension mHealth apps in terms of acceptance that complement the UTAUT determinants. In patients, protection motivation and perceived vulnerability made a significant explanatory contribution and should be thus further considered in efforts aimed at promoting mHealth acceptance. Furthermore, a deeper understanding of the underlying mechanisms in the theoretical model has been achieved by confirming performance expectancy as the mediator of the beliefs related to and intention to use mHealth apps.

## Appendix

Multimedia Appendix 1 Questionnaire of the German online survey (including translation)–patient and physician versions.

Multimedia Appendix 2 Linear model and influence of the unified theory of acceptance and use of technology predictors on utilization in patients (n=163).

Multimedia Appendix 3 Linear model and influence of protection motivation theory predictors on patients’ intention to use health apps (n=163).

## Abbreviations

DiGA: Digitale Gesundheitsanwendungen (German term for digital health applications)
eHEALS: electronic health literacy scale
mHealth: mobile health
PMT: protection motivation theory
UTAUT: unified theory of acceptance and use of technology
