# Supplementary material for: Comparing the Acceptance of Mobile Hypertension Apps for Disease Management Among Patients Versus Clinical Use Among Physicians: Cross-sectional Survey
Source: JMIR Cardio. 2022 Jan 6;6(1):e31617. doi: 10.2196/31617 (PMC8778565; doi:10.2196/31617)
Supplement: Multimedia Appendix 1 [file cardio_v6i1e31617_app1.docx]

Multimedia Appendix 1

**Questionnaire of the German online survey – patient and physician version**

The German version has been translated to English by a research team member for publication purposes only.

Content

[Patientenfragebogen (German) 2](#_Toc82262544)

[Ärztefragebogen (German) 6](#_Toc82262545)

[Patient questionnaire 9](#_Toc82262546)

[Physician questionnaire 12](#_Toc82262547)

## Patientenfragebogen (German)

1. **Demografische Faktoren (Kontrollvariablen)**
   - Alter (*metrisch*)
   - Geschlecht (*nominal*: 1 = weiblich, 2 = männlich, 3 = andere)
   - Höchster Bildungsabschluss (*ordinal*: 1 = (noch) Schüler, 2 = Von der Schule abgegangen ohne Abschluss, 3 = Hauptschulabschluss, 4 = Realschulabschluss (mittlere Reife), 5 = Fachhochschulreife, 6 = Allgemeine Hochschulreife (Abitur), 7 = Bachelor-Abschluss, 8 = Master/Diplom/Magister, 9 = Promotion, 10 = Habilitation)
   - Wohnsitz (*nominal:* 1 = Deutschland, 2 = Österreich, 3 = Schweiz, 4 = Anderes)
   - Wohnort (*nominal*: 1 = ländliche Region, 2 = städtische Region)
   - Beschäftigungsstatus (*nominal*: 1 = berufstätig in Vollzeit, 2 = berufstätig Teilzeit, 3 = (früh-)berentet, 4 = arbeitslos)
   - Haben Sie einen gesundheitsfachlichen- oder psychologischen Hintergrund (abgeschlossene Lehre/Ausbildung oder ein Studium)? (*nominal*: 1 = ja, 2 = nein)
   - Sind Sie aktuell als Fachkraft im Gesundheitswesen tätig? (*nominal*: 1 = ja, 2 = nein)
2. **Selbstwirksamkeit**

*(5-stufige Likert-Skala von „trifft voll und ganz zu“ bis „trifft überhaupt nicht zu“)*

- - In schwierigen Situationen kann ich mich auf meine Fähigkeiten verlassen.
  - Die meisten Probleme kann ich aus eigener Kraft gut meistern.
  - Auch anstrengende und komplizierte Aufgaben kann ich in der Regel gut lösen

1. **eHealth-Literacy** nach Soellner et al., 2014

*(5-stufige Likert-Skala von „trifft voll und ganz zu“ bis „trifft überhaupt nicht zu“)*

- - Ich weiß, wie ich im Internet nützliche Gesundheitsinformationen finde.
  - Ich weiß, wie ich das Internet nutzen kann, um Antworten auf meine Fragen rund um das Thema Gesundheit zu bekommen.
  - Ich weiß, welche Quellen für Gesundheitsinformationen im Internet verfügbar sind.
  - Ich weiß, wo im Internet ich nützliche Gesundheitsinformationen finden kann.
  - Ich weiß, wie ich Informationenaus dem Internet so nutzen kann, dass sie mir weiterhelfen.
  - Ich bin in der Lage, Informationen, die ich im Internet finde, kritisch zu bewerten.
  - Ich kann im Internet zuverlässige von fragwürdigen Informationen unterscheiden.
  - Wenn ich gesundheitsbezogene Entscheidungen auf Basis von Informationen aus dem Internet treffe, fühle ich mich dabei sicher.

1. **eHealth-Erfahrung**
   - Ich habe bereits Erfahrung mit Gesundheits-Apps (*nominal*: 1 = ja, 2 = nein)
   - Ich nutze Gesundheits-Appsn (Apps) für (*nominal* plus Freitext welche App, Mehrfachnennung möglich: 1 = Vitalwertmessung (z. B. Blutdruckkontrolle), 2 = Erinnerungen (z. B. an Medikation), 3 = Dokumentation, 4 = Entspannung, 5 = Recherche / Suche nach, 6 = Sonstiges, und zwar)
   - Diese Apps habe ich ausgewählt aufgrund (*nominal, Mehrfachnennung möglich: 1 =* Selbst gesucht / gefunden, 2 = Empfehlung von Freunden / Verwandten / Arbeitskollegen, 3 = Werbung, 4 = Sonstiges)
   - Ich nutze Gesundheits-Apps seit X Jahren (*metrisch*)
   - Ich suche Gesundheitsinformationen im Netz (privat) (*nominal:* 1 = ja, 2 = nein)
   - Ich suche Gesundheitsinformationen im Netz (beruflich) (*nominal:* 1 = ja, 2 = nein)
   - Im Internet suche ich bei gesundheitsbezogenene Informationen nach Informationen spezifisch zu (m)einer Erkrankung (*nominal:* 1 = ja, 2 = nein)
2. **Wahrgenommene Bedrohung der Privatsphäre** nach Zhang (2019)

*(5-stufige Likert-Skala von „trifft voll und ganz zu“ bis „trifft überhaupt nicht zu“)*

- - Ich denke, dass meine persönlichen Daten für andere Zwecke verwendet werden, wenn ich Gesundheits-Apps verwende.
  - Aus Sicherheitsgründen sehe ich mich dem Risiko des Verlustes personenbezogener Daten ausgesetzt, wenn ich Gesundheits-Apps verwende.
  - Ich denke, dass meine persönlichen Daten bei der Verwendung von Gesundheits-Appsn von Cyberkriminellen missbraucht werden.

1. **UTAUT-2** nach Venkatesh 2012, dt. Übersetzung nach Harborth 2018

*(5-stufige Likert-Skala von „stimme überhaupt nicht zu“ bis „stimme voll und ganz zu“)*

- Erwarteter Nutzen
  - Ich empfinde Gesundheits-Apps in meinem Alltag als nützlich.
  - Die Nutzung von Gesundheits-Apps erhöht meine Chancen, Dinge zu erreichen, die mir wichtig sind.
  - Die Nutzung von Gesundheits-Apps erhöht meine Produktivität.
- Erwartete Leichtigkeit der Bedienung
  - Zu lernen, wie Gesundheits-Apps genutzt werden ist einfach für mich.
  - Meine Interaktion mit Gesundheits-Apps ist klar und verständlich.
  - Ich finde, Gesundheits-Apps sind einfach zu nutzen.
  - Es ist einfach für mich, kompetent in der Nutzung von Gesundheits-Apps zu werden.
- Sozialer Einfluss
  - Personen, die mir wichtig sind, denken, ich sollte Gesundheits-Apps nutzen.
  - Personen, die mein Verhalten beeinflussen, denken, ich sollte Gesundheits-Apps nutzen.
  - Personen, deren Meinung ich schätze, denken, ich sollte Gesundheits-Apps nutzen.
- Erleichternde Bedingungen
  - Ich habe die notwendigen Ressourcen, um Gesundheits-Apps zu nutzen.
  - Ich habe das notwendige Wissen, um Gesundheits-Apps zu nutzen.
  - Gesundheits-Apps sind kompatibel mit anderen Technologien, die ich nutze.
  - Ich kann Hilfe von anderen bekommen, wenn ich Schwierigkeiten in der Nutzung von Gesundheits-Appsn habe.
- Hedonistische Motivation
  - eHealth-Anwendungen nutzen macht Spaß.
  - eHealth-Anwendungen zu nutzen ist vergnüglich.
  - eHealth-Anwendungen zu nutzen ist sehr unterhaltsam.
- Gewohnheit
  - Das Nutzen von eHealth-Anwendungen ist für mich zur Gewohnheit geworden.
  - Ich bin süchtig danach, eHealth-Anwendungen zu nutzen.
  - Ich muss eHealth-Anwendungen nutzen.

1. **Krankheitsbezug** (für alle Patienten)
   - Seit wie vielen Jahren wissen Sie von Ihrer chronischen Erkrankung (*metrisch*)
   - Seit wie vielen Jahren nehmen Sie Medikamente aufgrund Ihrer chronischen Erkrankung ein? (Wenn Sie keine Medikamente einnehmen, tragen Sie bitte „0“ ein. (*metrisch*)
   - Welchen Begleiterkrankungen haben Sie (1 = Herz-Kreislauf-Erkrankungen, 2 = Krebserkrankungen, 3 = chronische Lungenerkrankungen, 4 = Erkrankungen des Muskel-Skelett-Systems, 5 = psychische Störungen, 6 = Diabetes mellitus, 7 = andere)
2. **Schutzmotivationstheorie**

*(5-stufige Likert-Skala von „starke Zustimmung“ bis „starke Ablehnung“)*

**Mögliche Folgen eines hohen Blutdrucks sind unter anderem eine** Koronare Herzkrankheit, Herzschwäche, ein Herzinfarkt, ein Schlaganfall, pAVK, Netzhautschäden, Nierenschäden usw. Bitte bewerten Sie die folgenden Aussagen im Hinblick auf ihren hohen Blutdruck und mögliche Folgen.

- - Ich bin in Gefahr, die genannten Probleme zu haben
  - Es ist wahrscheinlich, dass ich die genannten Probleme haben werde.
  - Es ist einfach für mich, das angegebene Problem zu haben.
  - Wenn ich die genannten Probleme hätte, wäre es schwerwiegend.
  - Wenn ich die genannten Probleme habe, wäre es ernst.
  - Wenn ich die genannten Probleme habe, wäre es von erheblicher Bedeutung.
  - Gesundheits-Apps unterstützen mich bei der Lösung meines Problems.
  - Gesundheits-Apps sind bei der Lösung dieser Probleme effektiv.
  - Bei der Nutzung von Gesundheits-Apps ist die Lösung meiner Gesundheitsprobleme eher gewährleistet.
  - Es ist für mich einfach, Gesundheits-Apps zu nutzen.
  - Ich habe die Möglichkeit, Gesundheits-Apps zu nutzen.
  - Ich kann Gesundheits-Apps ohne großen Aufwand nutzen.

1. **Nutzungsintention nur für Patienten (Akzeptanz) nach** Hennemann et al. (2016)

*(5-stufige Likert-Skala von „starke Zustimmung“ bis „starke Ablehnung“)*

- - Gesundheits-Apps würde ich gerne ausprobieren.
  - Gesundheits-Apps würde ich nutzen, wenn es so etwas gibt
  - Gesundheits-Apps wäre es mir wert, dafür auch etwas zu bezahlen.

1. **Präferenz nur für Patienten (Akzeptanz)**

Aufgrund meines Bluthochdrucks würde ich am liebsten folgende Option in Anspruch nehmen (Einfachauswahl, *nominal*: 1 = Gesundheits-Apps speziell für Bluthochdruck, 2 = Webseiten zum Thema Bluthochdruck, 3 = Lokale Gruppen, 4 = Ärztliche Betreuung (Face-to-Face), 5 = Ärztliche Betreuung (via Internet), 6 = Selbsthilfe-Literatur, 7 = Andere)

## Ärztefragebogen (German)

1. **Demografische Faktoren (Kontrollvariablen)**
   - Alter (*metrisch*)
   - Geschlecht (*nominal*: 1 = weiblich, 2 = männlich, 3 = andere)
   - Höchster Bildungsabschluss (*ordinal*: 1 = (noch) Schüler, 2 = Von der Schule abgegangen ohne Abschluss, 3 = Hauptschulabschluss, 4 = Realschulabschluss (mittlere Reife), 5 = Fachhochschulreife, 6 = Allgemeine Hochschulreife (Abitur), 7 = Bachelor-Abschluss, 8 = Master/Diplom/Magister, 9 = Promotion, 10 = Habilitation)
   - Wohnsitz (*nominal:* 1 = Deutschland, 2 = Österreich, 3 = Schweiz, 4 = Anderes)
   - Wohnort (*nominal*: 1 = ländliche Region, 2 = urbane Region)
   - Beschäftigungsstatus (*nominal*: 1 = berufstätig in Vollzeit, 2 = berufstätig Teilzeit, 3 = (früh-)berentet, 4 = arbeitslos)
   - Fachrichtung (nominal plus Freitext: 1 = Innere Medizin, 2 = Allgemeinmedizin, 3 = Chirurgie, 4 = Anästhesiologie, 5 = Frauenheilkunde / Geburtshilfe, 6 = Kinder- und Jugendmedizin, 7 = Psychiatrie und Psychotherapie, 8 = Radiologie, 9 = Augenheilkunde, 10 = Neurologie, 11 = Hals-Nasen-Ohrenheilkunde, 12 = Urologie, 13 = Haut- und Geschlechtskrankheiten)
   - Berufserfahrung: Seit wie vielen Jahren sind Sie als Arzt tätig? (metrisch)
2. **Selbstwirksamkeit**

*(5-stufige Likert-Skala von „trifft voll und ganz zu“ bis „trifft überhaupt nicht zu“)*

- - In schwierigen Situationen kann ich mich auf meine Fähigkeiten verlassen.
  - Die meisten Probleme kann ich aus eigener Kraft gut meistern.
  - Auch anstrengende und komplizierte Aufgaben kann ich in der Regel gut lösen

1. **eHealth-Literacy** nach Soellner et al., 2014

*(5-stufige Likert-Skala von „trifft voll und ganz zu“ bis „trifft überhaupt nicht zu“)*

- - Ich weiß, wie ich im Internet nützliche Gesundheitsinformationen finde.
  - Ich weiß, wie ich das Internet nutzen kann, um Antworten auf meine Fragen rund um das Thema Gesundheit zu bekommen.
  - Ich weiß, welche Quellen für Gesundheitsinformationen im Internet verfügbar sind.
  - Ich weiß, wo im Internet ich nützliche Gesundheitsinformationen finden kann.
  - Ich weiß, wie ich Informationenaus dem Internet so nutzen kann, dass sie mir weiterhelfen.
  - Ich bin in der Lage, Informationen, die ich im Internet finde, kritisch zu bewerten.
  - Ich kann im Internet zuverlässige von fragwürdigen Informationen unterscheiden.
  - Wenn ich gesundheitsbezogene Entscheidungen auf Basis von Informationen aus dem Internet treffe, fühle ich mich dabei sicher.

1. **eHealth-Erfahrung**
   - Ich habe bereits Erfahrung mit Gesundheits-Apps (*nominal*: 1 = ja, 2 = nein)
   - Ich nutze Gesundheits-Apps für (*nominal* plus Freitext welche App, Mehrfachnennung möglich: 1 = Vitalwertmessung (z. B. Blutdruckkontrolle), 2 = Erinnerungen (z. B. an Medikation), 3 = Dokumentation, 4 = Entspannung, 5 = Recherche / Suche nach, 6 = Sonstiges, und zwar)
   - Diese Apps habe ich ausgewählt aufgrund (*nominal, Mehrfachnennung möglich: 1 =* Selbst gesucht / gefunden, 2 = Empfehlung von Freunden / Verwandten / Arbeitskollegen, 3 = Werbung, 4 = Sonstiges)
   - Ich nutze Gesundheits-Apps seit X Jahren (*metrisch*)
   - Ich suche Gesundheitsinformationen im Netz (privat) (*nominal:* 1 = ja, 2 = nein)
   - Ich suche Gesundheitsinformationen im Netz (beruflich) (*nominal:* 1 = ja, 2 = nein)
   - Im Internet suche ich bei gesundheitsbezogenen Informationen nach Informationen spezifisch zu (m)einer Erkrankung (*nominal:* 1 = ja, 2 = nein)
2. **Wahrgenommene Bedrohung der Privatsphäre** nach Zhang (2019)

*(5-stufige Likert-Skala von „trifft voll und ganz zu“ bis „trifft überhaupt nicht zu“)*

- - Ich denke, dass meine persönlichen Daten für andere Zwecke verwendet werden, wenn ich Gesundheits-Apps verwende.
  - Aus Sicherheitsgründen sehe ich mich dem Risiko des Verlustes personenbezogener Daten ausgesetzt, wenn ich Gesundheits-Apps verwende.
  - Ich denke, dass meine persönlichen Daten bei der Verwendung von Gesundheits-Apps von Cyberkriminellen missbraucht werden.

1. **UTAUT-2** nach Venkatesh 2012, dt. Übersetzung nach Harborth 2018

*(5-stufige Likert-Skala von „stimme überhaupt nicht zu“ bis „stimme voll und ganz zu“)*

- Erwarteter Nutzen
  - Ich empfinde Gesundheits-Apps in meinem Alltag als nützlich.
  - Die Nutzung von Gesundheits-Apps erhöht meine Chancen, Dinge zu erreichen, die mir wichtig sind.
  - Die Nutzung von Gesundheits-Apps erhöht meine Produktivität.
- Erwartete Leichtigkeit der Bedienung
  - Zu lernen, wie Gesundheits-Apps genutzt werden ist einfach für mich.
  - Meine Interaktion mit Gesundheits-Apps ist klar und verständlich.
  - Ich finde, Gesundheits-Apps sind einfach zu nutzen.
  - Es ist einfach für mich, kompetent in der Nutzung von Gesundheits-Apps zu werden.
- Sozialer Einfluss
  - Personen, die mir wichtig sind, denken, ich sollte Gesundheits-Apps nutzen.
  - Personen, die mein Verhalten beeinflussen, denken, ich sollte Gesundheits-Apps nutzen.
  - Personen, deren Meinung ich schätze, denken, ich sollte Gesundheits-Apps nutzen.
- Erleichternde Bedingungen
  - Ich habe die notwendigen Ressourcen, um Gesundheits-Apps zu nutzen.
  - Ich habe das notwendige Wissen, um Gesundheits-Apps zu nutzen.
  - Gesundheits-Apps sind kompatibel mit anderen Technologien, die ich nutze.
  - Ich kann Hilfe von anderen bekommen, wenn ich Schwierigkeiten in der Nutzung von Gesundheits-Apps habe.
- Hedonistische Motivation
  - Gesundheits-Apps nutzen macht Spaß.
  - Gesundheits-Apps zu nutzen ist vergnüglich.
  - Gesundheits-Apps zu nutzen ist sehr unterhaltsam.
- Gewohnheit
  - Das Nutzen von Gesundheits-Apps ist für mich zur Gewohnheit geworden.
  - Ich bin süchtig danach, Gesundheits-Apps zu nutzen.
  - Ich muss Gesundheits-Apps nutzen.

1. **Berufliche Nutzung des Smartphones analog zu Albrecht 2017**
   - Nutzen Sie ein Smartphone? (Nominal: 1 = Ja, 2 = Nein)
   - Zu welchem Zweck haben Sie Ihr Smartphone gekauft bzw. erhalten? (*Nominal*, Mehrfachnennung möglich: 1 = Ich habe ein oder mehrere Geräte für den privaten Gebrauch erworben / erhalten, 2 = Ich habe ein oder mehrere Geräte für den dienstlichen Gebrauch erworben / erhalten)
   - Für welche beruflichen Tätigkeiten nutzen Sie das Smartphone? (*Nominal*: 1 = Elektronische Kommunikation (E-Mail, Chat); 2 = Hinweise (Medikamente, Behandlungsoptionen), 3 = Literatursuche (Zeitschriften, Datenbanken), 4 = Lerninhalte (CME, eLearning), 5 = Als diagnostische Hilfe (Patientenkontakt), 6 = Organisieren / Auswahl der Behandlung, 7 = Patientenaufklärung / -information, 8 = Zugriff auf Patientendaten / -akten, 9 = Als Unterstützung zur Anforderung von Labortests oder bildgebenden Untersuchungen, 10 = andere Zwecke)
   - Was würde Sie davon abhalten, das Smartphone im Umgang mit Patienten zu benutzen? (*Nominal*: 1 = Bedenken hinsichtlich der Sicherheit von Patientendaten, 2 = Bedenken hinsichtlich der technischen Zuverlässigkeit des Smartphones, 3 = Bedenken hinsichtlich der technischen Zuverlässigkeit der Software, 4 = Bedenken hinsichtlich der Vertrauenswürdigkeit der Inhalte, die in der Software angezeigt werden, 5 = Bedenken hinsichtlich Hygiene, 6 = Patienten haben keinen Zugang zu einer solchen Technologie, 7 = Es ist nicht möglich, eine Kostenerstattung zu erhalten, 8 = Patienten akzeptieren diese Technology nicht, 9 = Keine Zeit oder nicht interessiert, 10 = Ich besitze kein Smartphone und plane auch nicht, eins zu kaufen, 11 = Andere)
2. **Abhängige Variable: Nutzungsintention (Akzeptanz)** analog zu Hennemann (2017)

*(5-stufige Likert-Skala von „starke Zustimmung“ bis „starke Ablehnung“)*

- 1. Ich könnte mir vorstellen, Gesundheits-Apps in meine Arbeit einzubeziehen.
  2. Ich beabsichtige, Gesundheits-Apps in meiner Arbeit innerhalb der nächsten 3 Monate auszuprobieren.
  3. Ich würde unseren Patienten eine Online-Nachbehandlung empfehlen.
  4. Wenn vorhanden, könnte ich mir vorstellen, einen Patienten mit Gesundheits-Apps zu betreuen.

## Patient questionnaire

1. **Demographic factors (control variables)**
   - Age (*metric*)
   - Gender (*nominal*: 1 = female, 2 = male, 3 = other)
   - Highest educational qualification (*ordinal*: 1 = (still) student, 2 = left school without qualification, 3 = secondary school qualification (basic), 4 = secondary school qualification (intermediate level), 5 = technical college entrance qualification, 6 = general university entrance qualification (German “Abitur”), 7 = bachelor's degree, 8 = Master / Diploma / Magister degree, 9 = Doctorate, 10 = Habilitation)
   - Residence (*nominal*: 1 = Germany, 2 = Austria, 3 = Switzerland, 4 = other)
   - Place of residence (*nominal*: 1 = rural region, 2 = urban region)
   - Employment status (*nominal*: 1 = employed full-time, 2 = employed part-time, 3 = (early) retired, 4 = unemployed)
   - Do you have a health or psychological (occupational) background (completed apprenticeship / training or a degree)? (*nominal*: 1 = yes, 2 = no)
   - Are you currently working as a healthcare professional? (*nominal*: 1 = yes, 2 = no)
2. **Self-efficacy**

*(5-point Likert scale from "strongly agree" to "strongly disagree".)*

- - I can rely on my abilities in difficult situations.
  - I can cope well with most problems on my own.
  - Usually, I can also solve strenuous and complicated tasks well.

1. **eHealth-Literacy** according to Norman and Skinner 2006

*(5-point Likert scale from "strongly agree" to "strongly disagree".)*

- - I know how to find helpful health resources on the Internet.
  - I know how to use the Internet to answer my health questions.
  - I know what health resources are available on the Internet.
  - I know where to find helpful health resources on the Internet.
  - I know how to use the health information I find on the Internet to help me.
  - I have the skills I need to evaluate the health resources I find on the Internet.
  - I can tell high quality from low quality health resources on the Internet.

I feel confident in using information from the Internet to make health decisions.

1. **eHealth experience**

- I already have experience with health apps (*nominal*: 1 = yes, 2 = no)
- I use health apps for (nominal plus free text which app: 1 = vital signs measurement (e.g. blood pressure control), 2 = reminders (e.g. of medication), 3 = documentation, 4 = relaxation, 5 = Research / search for, 6 = other, namely)
- I chose these apps on the basis of (*nominal*, multiple answers possible: 1 = searched / found myself, 2 = recommendation from friends / relatives / work colleagues, 3 = advertising, 4 = other)
- I have been using health apps for X years (*metric*)
- I am looking for health information on the internet (private) (*nominal*: 1 = yes, 2 = no)
- I am looking for health information on the internet (at work) (*nominal*: 1 = yes, 2 = no)
- On the Internet, I look for health-related information specific to (my) diseases (*nominal*: 1 = yes, 2 = no)

1. **Perceived privacy risk** nach Zhang (2019)

*(5-point Likert scale from "strongly agree" to "strongly disagree".)*

- - I think my personal privacy information will be used for other purposes if I use health apps.
  - Because of security issues, I face the risk of personal information leakage if I use health apps.
  - I think that when I use health apps, my personal information will be abused by cyber criminals.

1. **UTAUT-2,** based on Venkatesh 2012

*(5-point Likert scale from "strongly disagree" to "strongly agree")*

- - **Performance expectancy (expected benefits)**
    - I find health apps useful in my everyday life.
    - Using health apps increases my chances of achieving things that are important to me.
    - Using health apps increases my productivity.
  - **Effort expectancy (expected ease of use)**
    - Learning how to use health apps is easy for me.
    - My interaction with health apps is clear and understandable.
    - I think health apps are easy to use.
    - It is easy for me to become competent in the use of health apps.
  - **Social influence**
    - People who are important to me think I should use health apps.
    - People who influence my behavior think I should use health apps.
    - People whose opinions I value think I should use health apps.
  - **Facilitating conditions**
    - I have the necessary resources to use health apps.
    - I have the necessary knowledge to use health apps.
    - Health apps are compatible with other technologies that I use.
    - I can get help from others if I have difficulties using health apps.
  - **Hedonistic motivation**
    - Using health apps is fun.
    - Using health apps lications is pleasant.
    - Using health apps lications is very entertaining.
  - **Habit**
    - Using health apps has become a habit for me.
    - I am addicted to using health apps.
    - I have to use health apps.

1. **Experience related to illness** (for all patients)
   - How many years do you know about your chronic illness? (*metric*)
   - How many years have you been taking medication for your chronic illness? (If you are not taking any medication, please enter "0". (m*etric*)
   - Which concomitant diseases do you have (1 = cardiovascular diseases, 2 = cancer, 3 = chronic lung diseases, 4 = diseases of the musculoskeletal system, 5 = mental disorders, 6 = diabetes mellitus, 7 = other)
2. **Protection Motivation Theory**

*(5-point Likert scale from "strongly agree" to "strongly disagree".)*

Possible consequences of high blood pressure include coronary artery disease, heart failure, heart attack, stroke, PAOD, retinal damage, kidney damage, etc.

- - I am at risk of having the problems mentioned
  - It is likely that I will have the problems mentioned.
  - It is easy for me to have the stated problem.
  - If I had the problems mentioned, it would be severe.
  - If I have the problems mentioned, it would be serious.
  - If I have the problems mentioned, it would be of considerable importance.
  - eHealth offers support me in solving my problem.
  - eHealth services are effective in solving these problems.
  - When using health apps, the solution to my health problems is more likely to be guaranteed.
  - It is easy for me to use health apps.
  - I have the opportunity to use health apps.
  - I can use health apps with little effort.

1. **Dependent variable: Intention to use - only for patients (acceptance)** according to Hennemann et al. (2016)

*(5-point Likert scale from "strongly agree" to "strongly disagree".)*

- - I would like to try eHealth offers.
  - I would use eHealth offers if such a service existed.
  - It would be worth paying for eHealth services.

1. **Preference for patients only (acceptance)**
   - Because of my hypertension, I would prefer to use the following option (*single choice* (*nominal*: 1 = health apps specifically for hypertension, 2 = hypertension websites, 3 = local groups, 4 = medical care (face-to-face), 5 = medical care (via internet), 6 = self-help literature, 7 = other).

## Physician questionnaire

1. **Demographic factors (control variables)**
   - Age (*metric*)
   - Gender (*nominal*: 1 = female, 2 = male, 3 = other)
   - Highest educational qualification (*ordinal*: 1 = (still) student, 2 = left school without qualification, 3 = secondary school qualification (basic), 4 = secondary school qualification (intermediate level), 5 = technical college entrance qualification, 6 = general university entrance qualification (German “Abitur”), 7 = bachelor's degree, 8 = Master / Diploma / Magister degree, 9 = Doctorate, 10 = Habilitation)
   - Residence (*nominal*: 1 = Germany, 2 = Austria, 3 = Switzerland, 4 = other)
   - Place of residence (*nominal*: 1 = rural region, 2 = urban region)
   - Employment status (*nominal*: 1 = employed full-time, 2 = employed part-time, 3 = (early) retired, 4 = unemployed)
   - Do you have a health or psychological (occupational) background (completed apprenticeship / training or a degree)? (*nominal*: 1 = yes, 2 = no)
   - Are you currently working as a healthcare professional? (*nominal*: 1 = yes, 2 = no)
2. **Self-efficacy**

*(5-point Likert scale from "strongly agree" to "strongly disagree".)*

- - I can rely on my abilities in difficult situations.
  - I can cope well with most problems on my own.
  - Usually, I can also solve strenuous and complicated tasks well.

1. **eHealth-Literacy** according to Norman and Skinner 2006

*(5-point Likert scale from "strongly agree" to "strongly disagree".)*

- - I know how to find helpful health resources on the Internet.
  - I know how to use the Internet to answer my health questions.
  - I know what health resources are available on the Internet.
  - I know where to find helpful health resources on the Internet.
  - I know how to use the health information I find on the Internet to help me.
  - I have the skills I need to evaluate the health resources I find on the Internet.
  - I can tell high quality from low quality health resources on the Internet.

I feel confident in using information from the Internet to make health decisions.

1. **eHealth experience**

- I already have experience with health apps (*nominal*: 1 = yes, 2 = no)
- I use health apps for (nominal plus free text which app: 1 = vital signs measurement (e.g. blood pressure control), 2 = reminders (e.g. of medication), 3 = documentation, 4 = relaxation, 5 = Research / search for, 6 = other, namely)
- I chose these apps on the basis of (*nominal*, multiple answers possible: 1 = searched / found myself, 2 = recommendation from friends / relatives / work colleagues, 3 = advertising, 4 = other)
- I have been using health apps for X years (*metric*)
- I am looking for health information on the internet (private) (*nominal*: 1 = yes, 2 = no)
- I am looking for health information on the internet (at work) (*nominal*: 1 = yes, 2 = no)
- On the Internet, I look for health-related information specific to (my) diseases (*nominal*: 1 = yes, 2 = no)

1. **Perceived privacy risk** according to Zhang (2019)

*(5-point Likert scale from "strongly agree" to "strongly disagree".)*

- - I think my personal privacy information will be used for other purposes if I use health apps.
  - Because of security issues, I face the risk of personal information leakage if I use health apps.
  - I think that when I use health apps, my personal information will be abused by cyber criminals.

1. **UTAUT-2,** based on Venkatesh 2012

*(5-point Likert scale from "strongly disagree" to "strongly agree")*

- - **Performance expectancy (expected benefits)**
    - I find health apps useful in my everyday life.
    - Using health apps increases my chances of achieving things that are important to me.
    - Using health apps increases my productivity.
  - **Effort expectancy (expected ease of use)**
    - Learning how to use health apps is easy for me.
    - My interaction with health apps is clear and understandable.
    - I think health apps are easy to use.
    - It is easy for me to become competent in the use of health apps.
  - **Social influence**
    - People who are important to me think I should use health apps.
    - People who influence my behavior think I should use health apps.
    - People whose opinions I value think I should use health apps.
  - **Facilitating conditions**
    - I have the necessary resources to use health apps.
    - I have the necessary knowledge to use health apps.
    - Health apps are compatible with other technologies that I use.
    - I can get help from others if I have difficulties using health apps.
  - **Hedonistic motivation**
    - Using health apps is fun.
    - Using health apps is pleasant.
    - Using health apps is very entertaining.
  - **Habit**
    - Using health apps has become a habit for me.
    - I am addicted to using health apps.
    - I have to use health apps.

1. **Skala für Ärzte analog zu Albrecht 2017**
   - Do you use a smartphone? (*Nominal*: 1 = Yes, 2 = No)
   - For what purpose did you buy or receive your smartphone? (*Nominal*, multiple answers possible: 1 = I purchased / received one or more devices for private use, 2 = I purchased / received one or more devices for business use).
   - For which professional activities do you use the smartphone? (*Nominal*: 1 = Electronic communication (email, chat); 2 = Guidance (medications, treatment options), 3 = Literature search (journals, databases), 4 = Learning content (CME, eLearning), 5 = As a diagnostic aid (patient contact), 6 = Organizing / selecting treatment, 7 = Patient education / information, 8 = Accessing patient data / records, 9 = As a support to request lab tests or imaging studies, 10 = Other purposes).
   - What would prevent you from using the smartphone when interacting with patients? (*Nominal*: 1 = Concerns about security of patient data, 2 = Concerns about technical reliability of smartphone, 3 = Concerns about technical reliability of software, 4 = Concerns about trustworthiness of content displayed in software, 5 = Concerns about hygiene, 6 = Patients do not have access to such technology, 7 = Unable to obtain reimbursement, 8 = Patients do not accept this technology, 9 = No time or not interested, 10 = I do not own a smartphone and do not plan to purchase one, 11 = Other)
2. **Dependent variable: Intention to use (acceptance)** analog zu Hennemann (2017)

*(5-point Likert scale from "strongly agree" to "strongly disagree".)*

- - I could see myself incorporating health apps into my work.
  - I intend to try health apps in my work within the next 3 months.
  - I would recommend online follow-up to our patients.
  - If available, I could see myself mentoring a patient with health app.
